# Zebrafish Kidney Phagocytes Utilize Macropinocytosis and Ca^2+^-Dependent Endocytic Mechanisms

**DOI:** 10.1371/journal.pone.0004314

**Published:** 2009-02-02

**Authors:** Claudia Hohn, Sang-Ryul Lee, Lesya M. Pinchuk, Lora Petrie-Hanson

**Affiliations:** Department of Basic Sciences, College of Veterinary Medicine, Mississippi State University, Mississippi State, Mississippi, United States of America; University of California Merced, United States of America

## Abstract

**Background:**

The innate immune response constitutes the first line of defense against invading pathogens and consists of a variety of immune defense mechanisms including active endocytosis by macrophages and granulocytes. Endocytosis can be used as a reliable measure of selective and non-selective mechanisms of antigen uptake in the early phase of an immune response. Numerous assays have been developed to measure this response in a variety of mammalian and fish species. The small size of the zebrafish has prevented the large-scale collection of monocytes/macrophages and granulocytes for these endocytic assays.

**Methodology/Principal Findings:**

Pooled zebrafish kidney hematopoietic tissues were used as a source of phagocytic cells for flow-cytometry based endocytic assays. FITC-Dextran, Lucifer Yellow and FITC-*Edwardsiella ictaluri* were used to evaluate selective and non-selective mechanisms of uptake in zebrafish phagocytes.

**Conclusions/Significance:**

Zebrafish kidney phagocytes characterized as monocytes/macrophages, neutrophils and lymphocytes utilize macropinocytosis and Ca^2+^-dependant endocytosis mechanisms of antigen uptake. These cells do not appear to utilize a mannose receptor. Heat-killed *Edwardsiella ictaluri* induces cytoskeletal interactions for internalization in zebrafish kidney monocytes/macrophages and granulocytes. The proposed method is easy to implement and should prove especially useful in immunological, toxicological and epidemiological research.

## Introduction

The zebrafish, one of the most popular animals of developmental biologists, is rapidly gaining ground as an infection and immunology model [1]–[3]. The ease of producing specific zebrafish mutants is an additional benefit of using this model for experimental immunology [4]. Fish possess a well-developed, non-specific innate immune system, and phagocytes play an important role in the fish defense against microorganisms [5]–[13]. Phagocytic function has been used as an immunological parameter to evaluate the health status and immune function of different fish species under diverse biotic and abiotic factors such as pollutants [14], diets [15], temperature [16], pathogens [5] and genetic variation [17]. Cells of the monocyte/macrophage and granulocyte lineage are important elements of the immune defense system. These cells take up and destroy non-self damaged or apoptotic cells. Macrophages present antigens to lymphocytes and produce cytokines. Multiple mechanisms of endocytosis are used by different cell types [18]. In teleosts, B cells are also capable of ingesting particles and killing pathogens [19]. Even though our main focus lies in the role of zebrafish monocytes/macrophages and granulocytes for disease control, we included data supporting and expanding the findings by Li et al (2006) [19] on the endocytic abilities of teleost B cells.

In seabream the capture of antigens by surface receptors, such as the mannose receptor (MR) and glucan receptor was reported [20], [21]. In mammals these receptors allow efficient delivery of antigen to the processing compartment via receptor-mediated endocytosis [22]. MR-dependent endocytosis can be assessed by fluorescein isothiocyanate-labeled dextran (FITC-DX) uptake and inhibited by EDTA, anti-mannose receptor mAbs or mannan, a natural ligand of the MR in mammals [23]. Antigens that fail to bind to cell surface receptors can still be taken up by fluid phase endocytosis but with a lower efficiency [24]. Fluid phase uptake can occur via the distinct mechanisms of micropinocytosis and macropinocytosis [24]. In mammals, macropinocytosis is a potent non-selective mechanism of antigen uptake limited to immature dendritic cells and their myeloid progenitors, and monocytes/macrophages activated by exogenous stimuli [25], [26]. The internalization of solutes by macropinocytosis is much more effective than other fluid-phase uptake mechanisms, particularly micropinocytosis mediated by clathrin-coated vesicles [26]. Lucifer Yellow (LY) is traditionally used to assess macropinocytosis [23], [27], [28]. Cytochalasin D (CCD) is a cell permeable mycotoxin that inhibits macropinocytosis by blocking the formation of microfilaments and microtubules, but has no significant effect on receptor-mediated endocytosis [24].

Little is known about the selective and non-selective mechanisms of antigen uptake in fish. Zebrafish have been established as a model for the infectious disease Enteric Septicemia of Catfish caused by the intracellular pathogen *Edwardsiella ictaluri* [29]. The described endocytic assay was used to investigate the mechanisms of uptake of *E. ictaluri*. The small size of zebrafish (3–5 cm) has precluded the routine isolation of homogenous suspensions of monocytes or neutrophils and the additional lack of phagocytic cell lines have delayed the development of flow cytometric phagocytic assays for the zebrafish model system [30]. We describe a method of measuring antigen uptake in zebrafish phagocytes that utilizes flow cytometry to separate major blood cell lineages of zebrafish kidney cells (site of multilineage hematopoiesis) [31]. We gated for cells of interest eliminating the need of purification by gradient procedures. The endocytic abilities of kidney phagocytic cells were analyzed using electronic gating.

The aim of this study was to investigate the uptake mechanisms of zebrafish anterior kidney phagocytes. To achieve this aim, we modified existing phagocytic assays using flow-cytometry.

## Materials and Methods

### Zebrafish care

Zebrafish were housed in the CVM-MSU specific pathogen free fish hatchery [32] our water temperature was regulated by submersible heaters and closely monitored at 28°C±1. Maintenance and propagation of fish were performed according to modified standard protocols [33] and are posted at: http://www.cvm.msstate.edu/zebrafish/index.html.

All experiments were approved by the Institutional Animal Care and Use Committee at Mississippi State University.

### Cell preparation

Zebrafish were anesthetized in MS-222 (pH 7) [33]. Kidneys where excised as described previously [34]. To obtain single kidney cells, published kidney cell suspension protocols were modified [35]. Ten whole kidneys were pooled in 1 ml tissue culture media (RPMI-1640 supplemented with 10% fetal bovine serum, 1% Glutamax-1). Cells were disrupted from the whole kidney tissue by pipetting the suspension repeatedly. Cell suspensions were passed through a 40 µm cell strainer, collected in a 50 ml conical tube and rinsed with 1 ml tissue culture media. This procedure yielded approximately 7×10^6^ mixed kidney cells per ml.

### Endocytosis assays

The ability of kidney macrophages/monocytes and granulocytes to endocytose FITC-DX 70, FITC-DX 40 (Sigma-Aldrich Inc., St. Louis, MO), LY (Invitrogen Corporation, Carlsbad, CA) or heat-killed FITC-*Edwardsiella ictaluri* (FITC-*E. ictaluri*) as well as uptake of FITC-DX 40 and LY in zebrafish lymphocytes was measured following published procedures for mammalian cells [23], [27]. One hundred microliters of kidney hematopoietic cell suspension per sample was incubated for 30 min or 1 hour at 30°C representing our zebrafish holding temperature to measure active endocytosis or at 4°C to determine background levels of endocytosis (negative control). The cell suspension was washed three times by centrifugation at 400 g for 5 min and resuspended in ice-cold phosphate buffered saline (PBS) and analyzed by flow cytometry. Initial experiments also included incubation at 37°C. To monitor the effects of the incubation temperatures on cell viability within the gated cells, we evaluated cell death using propidium iodide staining (PI) [36]. Three samples were incubated for 1 hour at 4°C, 30°C and 37°C. Cells were washed as described above and PI was added at 5 µl (stock = 200 µg/ml) per milliliter of cells before analysis. To determine the mechanism of endocytosis, three different inhibitors were used. Cells were incubated for 5 min in the presence of inhibitor prior to 30 min or 1 hour incubation with FITC-DX 70 (500 µg/ml), FITC-DX 40 (500 µg/ml), LY (10 µg/ml), or FITC-*E. ictaluri* (1.8×10^8^ cells/mL). To inhibit macropinocytosis and phagocytosis 2.5 µg/ml, Cytochalasin D (CCD) (Sigma-Aldrich Inc., St. Louis, MO) was used. To inhibit Ca^2+^-dependent endocytosis that is usually receptor-mediated, samples were incubated with EDTA (1 mM). Mannan (500 ug/ml) (Sigma-Aldrich Inc., St. Louis, MO) was added to inhibit specific uptake by the MR. Each endocytic assay was carried out in triplicate from a cell suspension of pooled kidney cells from 10 fish.

**Figure 1:**
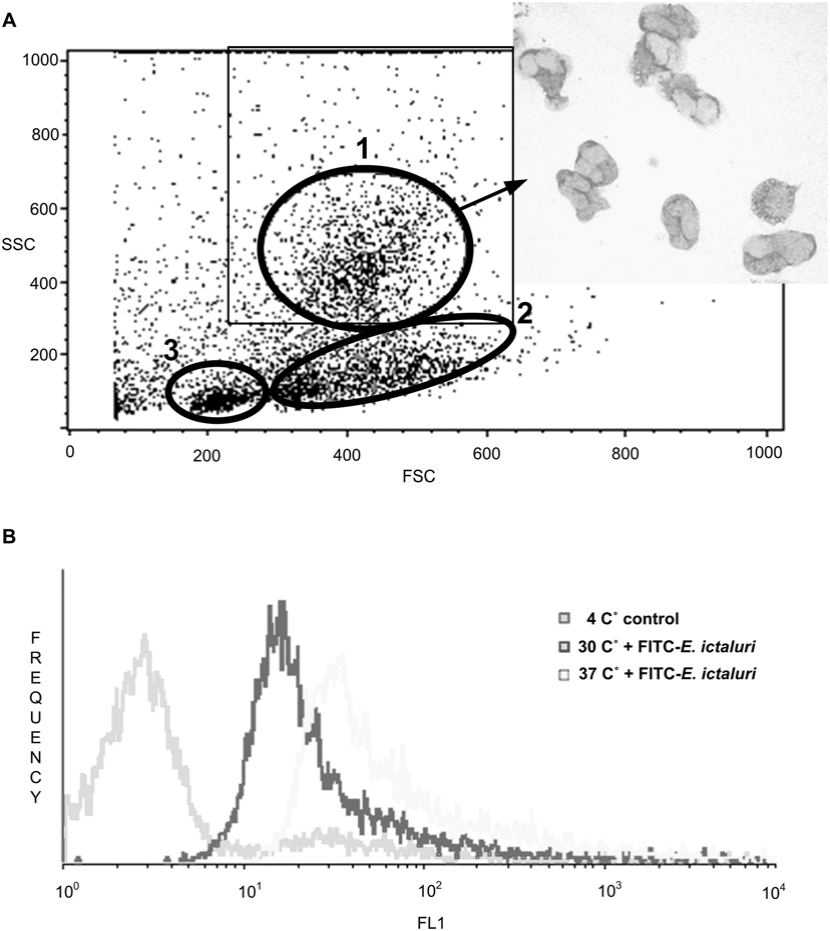
Electronic separation of kidney leukocytes by flow cytometry and measurement of fluorescence in phagocytic cells. A) Forward Scatter (FSC) and Side Scatter (SSC) characteristics of kidney cell suspensions differentiate 3 distinct cell populations in zebrafish 1: macrophage/monocytes and granulocytes, 2: hematopoietic precursors, 3: lymphocytes and lymphocyte-like cells. Inset shows Wright stain of sorted cells from gate 1. B) Endocytosis was assessed by measuring green fluorescent intensity (FL1) in the gated macrophage/monocytes and granulocytes or lymphocytes. The fluorescent peaks in this example indicate active macropinocytosis of FITC-*E. ictaluri* at 30°C and 37°C compared to 4°C control in gate1. Since ingestion of bacteria could alter size and granularity of cells the analytical gating for phagocytes in gate 1 was expanded (square gate) to control a potential shift of cells. Note about inset: Sorted cells image was taken separately from endocytosis experiments. The described method does not rely on actual cell sorting but rather on electronic gating of cell populations.

To study dose dependent uptake, kidney cells were incubated for 1 hour at 30°C with 4 different concentrations of FITC-DX 40 (100 µg/ml, 500 µl/ml, 1 mg/ml, 2 mg/ml PBS) or LY (0.25 µg/ml, 2.5 µg/ml, 12.5 µg/ml, 25 µg/ml). Cells in gate 1 (Figure 1A) where analyzed for endocytic uptake.

To test for appropriate dosages of the inhibitors CCD and EDTA as well as EtOH (the solvent for CCD), CCD inhibition of LY was performed at 3 concentrations: 2.5 µg/ml, 5 µg/ml, 10 µg/ml. In a parallel study the effect of EtOH (molecular grade, 200-proof) at 5 µl/ml, 10 µl/ml, 20 µl/ml, on kidney cells was tested. EDTA was added to FITC-*E. ictaluri* at: 1 mM, 5 mM, 10 mM. All dosage studies were conducted in triplicates at 30°C and results compared to untreated 4°C and 30°C controls.

### Labeling of *E. ictaluri* with FITC

*E. ictaluri* (93146 WT#19) was labeled with FITC following the *Vibrio anguillarum* labeling protocol of Chavez-Pozo *et al.* [37]. Bacteria were grown over-night in brain heart infusion (Becton Dickinson, Franklin Lakes, NJ) supplemented with 50 µg/ml FITC (Sigma-Aldrich Inc., St. Louis, MO) at 30°C in a light-protected environment. Bacteria were washed three times in PBS by centrifugation for 10 min at 1000g and killed by heating at 60°C for 20 min. After an additional washing step, optical density (OD) was measured and bacterial concentrations were adjusted to 1.8×10^8^ cells/ml (OD 0.4 at 540 nm) and used in endocytosis assays.

### Flow cytometry

To measure Mean Fluorescent Intensity (MFI), samples were mixed gently, acquired and analyzed by a FACS Calibur flow cytometer (Becton Dickinson, Franklin Lakes, NJ). Initially, the instrument settings were adjusted to obtain optimal separation of the different cell populations present in zebrafish kidney leukocytes [38], [39]. The data of a total of 200,000 cells per sample were collected with an average of 67,000 cells in gate 1 and 23,000 cells in gate 2 (Figure 1A). Data were analyzed as dot plots using Side scatter (granularity) (SSC) and Forward scatter (size) (FSC) parameters (Figure 1A). After setting an electronic gate on macrophages/monocytes and granulocytes, incorporation of FITC-DX, LY or FITC-*E. ictaluri* was measured as green fluorescence (FL1) at 530 nm (Figure 1B), expressed as MFI, and analyzed using CellQuest™ Pro software (Becton Dickinson, Franklin Lakes, NJ).

Cell sorting was performed in the flow cytometry core facility at the LSU Health Sciences Center in Shreveport. Kidney cells (Figure 1A) were sorted by FACSAria™ (Becton Dickinson, Franklin Lakes, NJ) to reproduce previous findings [31], to justify electronic gating used by the described phagocytosis assay and for photography.

### Statistical analysis

Fluorescence is represented as the mean±s.e.m.. All endocytosis assays were performed in triplicate. Statistical significance was determined using ANOVA with LSD correction for multiple comparisons as a post hoc test. Statistical significance was accepted at p≤0.05. Statistical analyses were performed using SPSS® for Windows 15.0 (SPSS Inc., Chicago, IL).

## Results

### Characterization of zebrafish leukocytes

When hematopoietic cells of zebrafish were evaluated by flow cytometry, three distinct non-erythrocyte cell populations were electronically separated according to their size (FSC) and granularity (SSC) (Figure 1A): 1) macrophage/monocytes and granulocytes, 2) hematopoietic precursors, and 3) lymphocytes and lymphocyte-like cells. In Figure 1B the MFI of the phagocytic cells in gate 1 is demonstrated. They formed a very distinct cell population, making electronic gating possible and eliminating the need to sort phagocytes from other cell populations. For the phagocytic assays, we sometimes observed a shift in phagocyte populations in gate 1. This shift was most likely due to uptake of particles during incubation and did not overlap with the other cell populations. To account for this change in size and granularity the electronic gate was expanded (Figure 1A).

### Viability and inhibitors

#### Propidium iodide (PI) control

**Figure 2:**
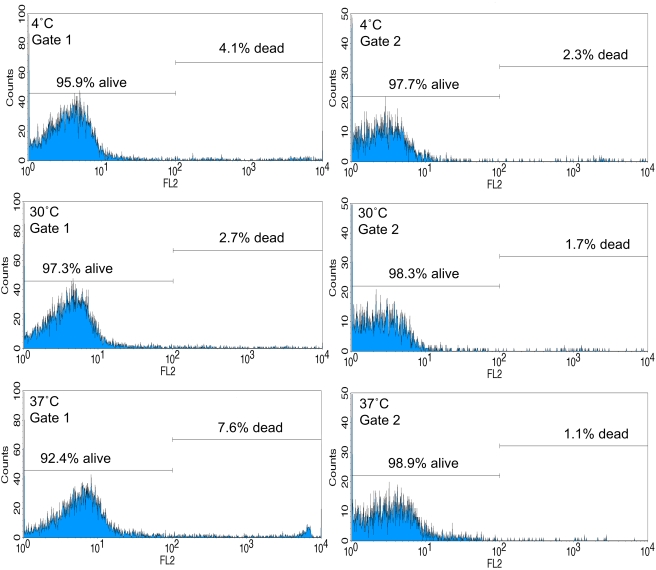
Cell death analyses by Propidium iodide (PI) fluorescence. Propidium iodide was used to monitor cell death at different incubation temperatures used in this study. Only dead cells take up PI and emit red fluorescence (FL2). Phagocytes (Gate1 Figure 1A) and lymphocytes (Gate 2 Figure 1A) were incubated for 1 hour at 4°C, 30°C and 37°C before addition of PI.

PI was added to cells that had been incubated at 4°C, 30°C and 37°C to monitor the possible adverse effect of the incubation temperatures on zebrafish kidney cells. Cells incubated at 30°C, close to our fish holding temperature, showed only a small percentage of dead cells; 97% of monocytes/macrophages and granulocytes and 98% of lymphocytes were viable. At 4°C alive cells also exceeded 95% in both gates. At 37°C a slight increase of necrotic cells with 92.4% viable cells was observed (Figure 2).

#### Dosage of inhibitors

CCD and EDTA were tested at different concentrations to establish working concentrations for maximum inhibition without toxic effects on cells. At a concentration of 2.5 µg CCD per milliliter EtOH, a 40% inhibition of LY uptake occurred. In a parallel study the corresponding concentration of 5 µl EtOH per ml of kidney cells showed no adverse effects on LY uptake. At higher CCD concentrations no increase in inhibition was observed whereas higher EtOH concentrations affected the endocytic ability of cells (data not shown).

A 30% inhibition of heat-killed FITC-*E. ictaluri* uptake due to EDTA (1 mM) was observed. Higher concentrations of EDTA did not further inhibit the uptake of bacteria (data not shown).

### Fluid-phase uptake and receptor-mediated endocytosis in zebrafish phagocytes

#### Non-selective uptake

**Figure 3:**
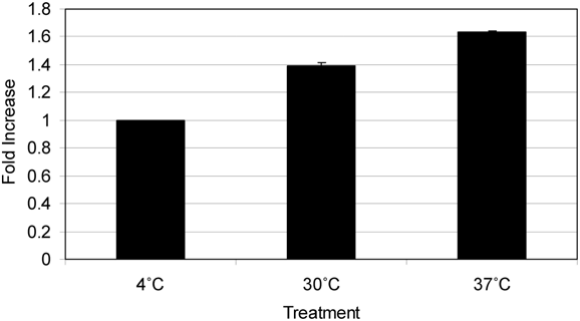
Non-selective uptake via macropinocytosis of Lucifer Yellow (LY) in zebrafish kidney phagocytes. The *y*-axis represents fold increase in mean fluorescent intensity (MFI) compared to basal conditions (4°C treatment). The *x*-axis represents the experimental conditions during incubation. A) After an incubation time of 30 min a significant uptake of LY at 30°C and 37°C was observed. B) The inhibition affect of Cytochalasin D (CCD) (2.5 µg/ml) when added to kidney cells prior to incubation for 1 hour with LY. Addition of the inhibitor EDTA (1 mM) had no significant effect on LY uptake in granulocytes and macrophages. C) Macropinocytosis in zebrafish lymphocyte population. Non-selective uptake of LY was also observed in cells of the lymphocyte gate. This uptake was significantly inhibited by CCD (2.5 µg/ml) but not by EDTA (1 mM). Same letters indicate no significant difference in MFI. Average fold change in MFI±s.e.m. from 3 replicates is shown (*p*<0.05).

**Figure 4:**
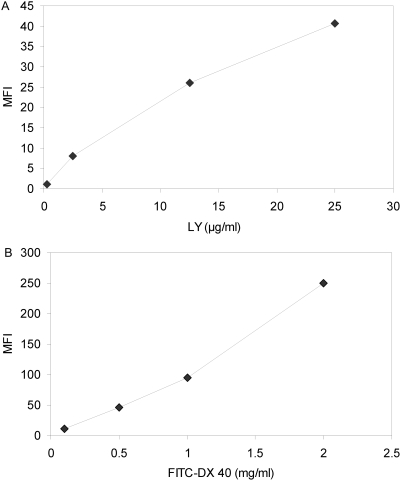
Endocytosis of different markers in renal monocytes/macrophages and granulocytes reveals nonsaturable mechanisms of uptake. Cells were incubated at 30°C in the presence of different concentrations of Lucifer Yellow (LY) (A) or FITC-DX 40 (B) and the amount accumulated was measured as mean fluorescent intensity (MFI) after 1 hour. The background fluorescence (cells incubated at 4°C) was subtracted. Both graphs show a dose dependent nonsaturable uptake indicative of macropinocytosis as mechanism of endocytosis.

Lucifer Yellow was actively taken up by zebrafish monocytes/macrophages and granulocytes at 30°C, and increased numerically at 37°C (Figure 3A). The increased uptake seen at 37°C (Figure 3A) was reproduced by prolonging the incubation times from 30 min to 1 hour (Figure 3B,C). The addition of Cytochalasin D (CCD), a potent inhibitor of actin polymerization, significantly inhibited LY uptake in zebrafish phagocytes whereas the addition of EDTA had no significant adverse effect on LY uptake (Figure 3B,C) suggesting macropinocytosis as the mechanism of uptake. Fluid phase uptake was not saturable with increasing concentrations of the antigen LY. The amount of LY accumulated by monocytes/macrophages and granulocytes was proportional to the concentration of LY in the medium, indicating macropinocytosis as the mechanism of uptake (Figure 4A).

We were also able to demonstrate fluid-phase uptake via macropinocytosis in zebrafish lymphocytes (3C). The uptake increased from 10.8±0.4 mean fluorescent intensity (MFI) at 4°C to 18.9±0.3 MFI at 30°C (Figure 3B). The lymphocytes did take up LY, but at a much lower amount per cell, with 3.8±0.6 MFI at 4°C to 8.4±0.2 MFI at 30°C (Figure 3C).

#### Mannose receptor-mediated antigen uptake

**Figure 5:**
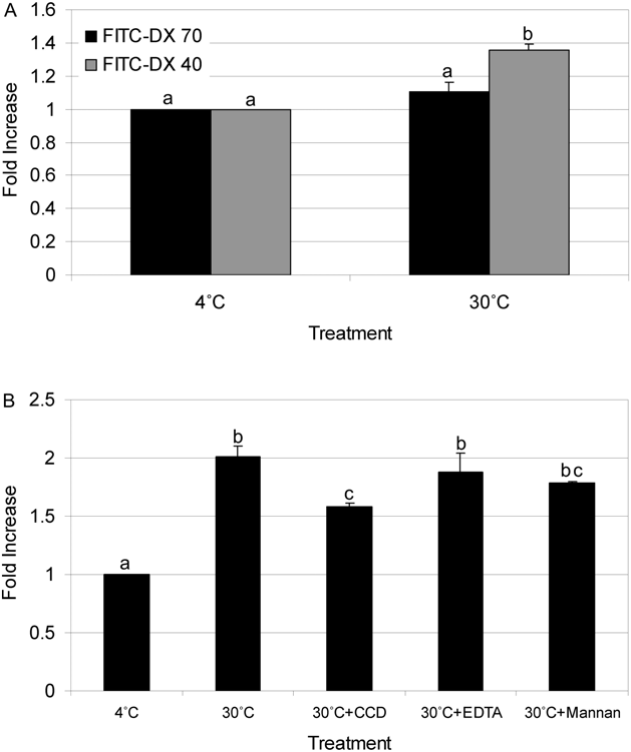
Uptake of FITC-DX by zebrafish kidney phagocytes. The *y*-axis represents fold increase in mean fluorescent intensity (MFI) compared to basal conditions (4°C treatment). The *x*-axis represents the experimental conditions during incubation. A) A comparison of uptake of FITC-DX 40 to FITC-DX 70 after 30 min at 30°C. B) The affect of Cytochalasin D (CCD), EDTA and Mannan on FITC-DX 40 uptake after 1 hour incubation at 30°C. Same letters indicate no significant difference in MFI. Average fold change in MFI±s.e.m. from 3 replicates is shown (*p*<0.05).

Incubation of zebrafish phagocytes with FITC-DX 70 showed no significant active uptake in antigen at 30°C or 37°C (Figure 5A). Incubation of zebrafish phagocytes with the smaller antigen FITC-DX 40 demonstrated a significant 1.35 fold increase in uptake over the 4°C control samples (Figure 5A). To get information about the mechanisms of uptake of FITC-DX 40, we used 3 different inhibitors and measured the amount taken up as a function of the concentration in solution. No significant inhibition was observed when EDTA or mannan was added. Partial inhibition occurred in the CCD treatment (Figure 5B). In addition, the amount of FITC-DX 40 accumulated by the phagocytes was unsaturable (Figure 4B). These findings suggest that uptake of FITC-DX in zebrafish kidney phagocytes is non-specific and that in this study, mannose receptor was not involved.

#### Mechanisms of heat-killed *E. ictaluri* uptake in zebrafish phagocytes

**Figure 6:**
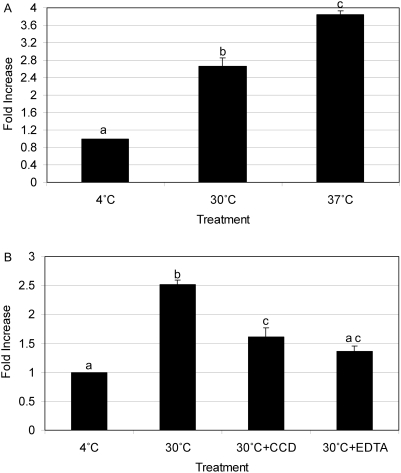
Uptake of heat-killed FITC-*E. ictaluri* in zebrafish kidney phagocytes. The *y*-axis represents fold increase in mean fluorescent intensity (MFI) compared to basal conditions (4°C treatment). The *x*-axis represents the experimental conditions during incubation. A) Significant uptake of FITC-*E. ictaluri* at 30°C and 37°C when compared to 4°C control treatment was demonstrated. B) Significant inhibition after incubation of kidney cells with CCD is shown indicating non-selective uptake via macropinocytosis. Adding EDTA also significantly inhibited FITC-*E. ictaluri* uptake. EDTA is known to inhibit receptor-mediated endocytosis. Same letters indicate no significant difference in MFI. Average fold change in MFI±s.e.m. from 3 replicates is shown (*p*<0.05).

Renal phagocytic cells were incubated with heat-killed FITC-*E. ictaluri* in the presence of the inhibitors CCD or EDTA and demonstrated significant active uptake of *E. ictaluri* at 30°C and a further significant increase in uptake at 37°C (Figure 6A). When cells were incubated with CCD, significant reduction of actin-dependent uptake was measured, indicating that macropinocytosis plays an important role in the internalization of this pathogen (Figure 6B). Further, when kidney phagocytes were incubated with FITC-*E. ictaluri* in the presence of EDTA, a significant inhibitory effect of EDTA suggested that Ca^2+^-dependant receptor-mediated endocytosis was also involved in the uptake of heat-killed *E. ictaluri* by zebrafish kidney phagocytes (Figure 6B).

## Discussion

With an average length of 3–5 cm and blood yield of 10 µl per zebrafish, commercially available phagocytosis test kits are not practical for use in zebrafish. Traditional tests using fluorescent or light microscopy to calculate phagocytic index or phagocytic capacity are labor intensive and can be biased [11], [40]–[42]. Studies in which microscopic assessment of phagocytosis in head kidney granulocytes of fresh water fish were compared to the flow cytometry method reported accurate correlations between procedures [43], [44]. Our findings also support flow cytometry as a suitable method for endocytic studies in fish. A well known difficulty of zebrafish immunological research is the paucity of mAbs against zebrafish blood cells, which excludes use of fluorescent activation cell sorting, a technique sometimes used in other fish species to sort for cells of interest [45]. Utilization of flow-cytometry to electronically gate for kidney cells of interest as first shown by Traver *et al.* (2003) [31] avoids the need to separate cell populations by gradients, which is difficult when small numbers of cells are being used. Additionally, electronic gates can be set at multiple cell populations of interest and data can be acquired simultaneously and electronic gates can be extended to compensate for morphological changes in cells due to uptake of particles. Utilizing flow-cytometry greatly reduces the number of animals used and at the same time allows replicates to be analyzed quickly to obtain reproducible data. Peripheral blood assays study phagocytosis in mammalian macrophages and neutrophils have been described [46]–[49] but erythrocyte populations complicate cell separations in zebrafish. Lysis of fish erythrocytes is more difficult compared to mammals [50], and results in nuclei that falsely alter leukocyte counts. Separation of unlysed, whole blood cell populations, utilizing the specific stains DiOC_6_ and DiOC_5_, has been demonstrated in common carp [51] with the potential to separate lymphocytes from thrombocytes [52]. Although this technique was unsuccessful in zebrafish whole blood, in zebrafish kidney cell preparations forward and side scatter properties did separate cell populations (Figure 1) [4], [31], [38], [39], and thrombocytes comprised only 0.5% of total leucocytes [34], [53].

Control samples incubated at 4°C indicated very little non-specific uptake or superficial adherence occurred. The fluorescence detected in the control sample corresponds to adherence and/or non-specific antigen uptake, which is not affected at low temperatures, while active cellular functions, including phagocytosis, are inhibited [48]. To validate the use of reagents and procedures, optimized for mammalian cells, we initially incubated zebrafish cells at 30°C and 37°C. We found that MFI was increased at 37°C and therefore prolonged the incubation time to offset the lower incubation temperature of fish cells.

Macropinocytosis is a major endocytic pathway involved in non-selective bulk fluid-phase uptake [18], and LY is a model antigen to investigate this mechanism of uptake in mammals [23], [54]. Our study demonstrates that zebrafish phagocytes are able to capture LY by macropinocytosis and like cattle [27], [55] and human dendritic cells [23], zebrafish phagocytes use macropinocytosis for a bulk-fluid uptake of soluble antigens. Li *et al.* [19] investigated the phagocytic abilities of B cells in trout and channel catfish. We utilized this flow cytometry based phagocytic assay to investigate endocytosis in zebrafish lymphocytes and we were able to show fluid phase uptake by macropinocytosis in gated populations of lymphocyte and lymphocyte-like cells. Future investigation will show if zebrafish lymphocytes are also capable of receptor mediated uptake of particles as has been shown in trout and *Xenopus laevis* [19].

Zebrafish are increasingly utilized as a model for human pathogens [56]–[59] as well as economically important fish pathogens [13], [29], [60]. Since tested compounds where successfully taken up at 30°C, closely representing the zebrafish holding temperature in our facility, and 37°C, the optimal temperature for mammalian pathogens, the data presented here suggests a possible application of zebrafish phagocytic cells in some mammalian disease models. Propidium iodide controls demonstrated the viability of zebrafish cells at all tested incubation temperatures. Dead cells have different scatter properties than living cells. In particular, because of their perforated outer membrane, they have a lower refractive index than living cells and therefore have forward scatter signals of lower intensity [61]. Therefore it is generally advised not to use gates when analyzing a population for the proportion of dead and live cells. Since we used electronic gating to measure endocytosis in different phagocyte populations we decided to also measure necrosis specifically within those gates. When using this phagocytic assay the forward and side scatter plot indicates the status of the cells since dead or dying cells are visible in the lower left of the scatter plot outside the electronic gates and are therefore not analyzed for phagocytosis.

Zebrafish have been evaluated as a model for *E. ictaluri* pathogenesis [29], and demonstrate characteristic pathology. In the current study, heat-killed FITC-*E. ictaluri* was used to investigate the mechanism of *E. ictaluri* uptake in zebrafish kidney phagocytic cells. Skirpstunas *et al*. used chemical inhibitors to demonstrate the importance of cellular microfilament and receptor-mediated endocytosis in the uptake of *E. ictaluri* in mammalian epithelial cells [62]. The inhibitor CCD specifically bound to actin causing microfilament depolymerization, which resulted in altered cell morphology and interference with bacterial adherence and entry [63]. EDTA blocks receptor mediated endocytosis which involves phagocytosis and micropinocytosis. The data presented here demonstrate that uptake requiring surface alterations to facilitate either adherence and/or internalization was substantially reduced after incubation with CCD and EDTA, suggesting that heat-killed *E. ictaluri* may also induce a system of cytoskeletal interactions for internalization in zebrafish kidney phagocytic cells.

FITC-DX is used to demonstrate MR-mediated endocytosis, and is accepted as a classical model antigen for mammalian antigen presenting cells [23], [27]. Many pathogens have abundant mannose, glucose and other sugars on their cell surfaces. Specific receptors recognize these sugars, and are well-characterized in mammals. The MR is the most studied of the lectin-like receptors [64], [65]. These receptors constitute an essential part of the host defense system because they are involved in phagocytosis of infectious agents and in the internalization of parasites that replicate inside phagocytes [66]. Fish possess the putative MR proteins that exhibit structural similarity to other vertebrate MR proteins, suggesting that they are present in all vertebrates [21]. In zebrafish the mannose 6-phosphate receptor has been biochemically characterized and the amino acid sequence was found to be 51% identical to human and 53% identical to chicken receptor [67], [68]. When expressed in mannose receptor deficient mouse embryonic cells, the zebrafish MR rescued phosphomannan binding [67]. In a separate study, four amino acid residues essential for carbohydrate recognition by the bovine MR were found to be conserved in zebrafish MR [68]. When zebrafish phagocytes were incubated with FITC-DX 70 no significant uptake occurred. The smaller FITC-DX 40 was taken up but inhibition with the MR specific inhibitor mannan was unsuccessful, suggesting uptake was due to macropinocytosis. Esteban *et al*. [20] reported an involvement of glucan receptor but not MR in the phagocytosis of pathogens by seabream peripheral blood leukocytes. However, Rodriguez *et al*. [21] indicated MR mediated uptake in seabream kidney phagocytes. In mammals, MR expression is minimal in immature bone marrow monocyte/macrophage but when induced to mature by immunoglobulin G (IgG) exposure MR surface expression is up regulated as much as 7- to 12-fold [69]. IgF binding sites have also been reported on trout MR [70]. Considering that we utilized zebrafish grown under specific pathogen free conditions and the cells used were from the bone marrow equivalent in fish, the possibility of low expression of MR in these cells could account for the lack of mannan inhibition and the apparent uptake of FITC-DX by macropinocytosis alone. Further investigations are needed to clarify these conflicting findings of MR mediated uptake in teleost kidney phagocytes.

In conclusion, the data presented here underlines the applicability of zebrafish in both fish and mammalian disease models. Zebrafish kidney phagocytes utilize macropinocytosis and Ca^2+^ dependant endocytic mechanisms. Furthermore, heat-killed *E. ictaluri* induces a complex system of cytoskeletal interactions for internalization in zebrafish kidney phagocytic cells. We speculate that MR mediated phagocytosis is not an important mechanism for pathogen recognition in zebrafish kidney cells. However, this receptor is likely important for pathogen recognition in differentiated macrophages. Flow cytometry is a quick, reproducible, and objective method to evaluate the endocytic capacity of zebrafish renal phagocytes. Additionally, this method is particularly useful for simultaneous multi-parameter analyses of small sample volumes and provides a simple and rapid assay for studying innate immunity in zebrafish. Flow cytometric endocytic assays will enable researchers to study the effects of pathogens, environmental toxins and stress on fish immune health utilizing the zebrafish model.
